# The relationship between college students' belief in a just world and internet addiction: the moderating effect of legal cognition

**DOI:** 10.3389/fpsyg.2025.1557781

**Published:** 2025-04-25

**Authors:** He Jianhua, Zhu Haoliang, Li Kanze, Xu Shuhui

**Affiliations:** ^1^School of Mechanical Engineering, Tongling University, Anhui, China; ^2^Department of Psychology, Wenzhou University, Wenzhou, China

**Keywords:** belief in a just world, internet addiction, legal cognition, college students, abstract legal cognition

## Abstract

**Background:**

Previous studies have explored various personality and emotional variables influencing internet addiction. However, few have examined the impact mechanism of legal cognition on internet addiction. This study examined the relationship between belief in a just world and internet addiction, investigating whether legal cognition could moderate the effect of belief in a just world on internet addiction.

**Methods:**

The study used the Belief in a Just World Scale, the Internet Addiction Scale, and the Legal Cognition Assessment Scale to survey 532 college students from universities in mainland China.

**Results:**

Correlation analysis indicated a significant negative correlation between belief in a just world and internet addiction; belief in a just world negatively predicted internet addiction, and both the overall score of legal cognition and abstract legal cognition moderated this relationship.

**Conclusions:**

College students' internet addiction was influenced by belief in a just world and legal cognition, suggesting that interventions for internet addiction among college students could involve cognitive-behavioral therapy and education on the rule of law.

## 1 Introduction

### 1.1 Belief in a just world and internet addiction

Internet addiction refers to the inability of individuals to control their use of the internet, resulting in harm to their physical and mental health and difficulties in social adaptation (Davis, 2001). It is a loss of control over behavior due to prolonged inappropriate internet use, which is accompanied by significant impairments in social and psychological functioning (Guo et al., 2018). Internet addiction adversely affects individuals' academic performance, work, emotions, and interpersonal relationships (Geng et al., 2023; Ivezaj et al., 2017; Xie et al., 2023; Gu et al., 2024). It also harms individuals' physical and mental health, leading to adverse outcomes such as decreased academic performance and social adaptation (Stanković et al., 2021; Davis, 2001; Iyitoglu and Çeliköz, 2017), and is even an important predictor of aggressive behavior (Peng et al., 2022). A survey of Chinese adolescents aged 12 to 18 showed that the prevalence of internet addiction reached 16.8% (Peng et al., 2022). Therefore, it is essential to explore the mechanisms underlying internet addiction among adolescents.

Currently, there are several theories that explain the reasons for internet addiction. The compensatory internet use model posits that individuals may compensate for unmet needs in real life by engaging in the online world (Kardefelt-Winther, 2014). This model is consistent with the uses and gratifications theory, which suggests that the reasons for adolescent internet gaming addiction lie in the rich game content and social interaction environments provided by online games. Adolescents can fully express themselves in games, greatly satisfying their self-esteem needs; thus, online gaming often becomes a primary means for individuals with low self-esteem to fulfill psychological needs and relieve emotional pressure (Yi and Li, 2006). A longitudinal study indicated that shyness was a prerequisite for internet addiction, while self-esteem was a consequence of internet addiction. Other findings also suggested that loneliness and depression were precursors to internet addiction (Tian et al., 2021). Internet addiction research initially focused on individuals who experienced relational problems, academic issues, financial difficulties, and unemployment due to their inability to control their internet use (Young, 1998). It is evident that internet addiction is indeed a form of maladaptive social adaptation, and it can be considered a coping mechanism for individuals facing life's difficulties. Perhaps individuals' cognition or beliefs about society or the world can be considered antecedent variables for research. Accordingly, this introduces the theme of the current study, which aims to explore the impact of belief in a just world on internet addiction.

Lerner proposed the just world hypothesis, which posits that individuals need to believe they live in a world where people generally get what they deserve. Belief in a just world leads individuals to perceive their material and social environment as stable and orderly. Without such beliefs, individuals may struggle to commit to pursuing long-term goals and may even find it difficult to engage in daily behaviors that align with social norms (Lerner, 1977). In other words, belief in a just world refers to the need for individuals to believe that they can receive what they deserve, allowing them to make sustained efforts toward a goal (Lerner and Miller, 1978). Research on belief in a just world typically distinguishes between personal belief in a just world (belief in a just world for oneself) and general belief in a just world (belief in a just world for others) (Sutton and Douglas, 2005). Personal belief in a just world typically reflects an individual's perception of whether they are treated fairly. Individuals with a strong personal belief in a just world tend to view the past and future positively. When faced with injustice, these individuals may reassess the situation and achieve cognitive harmony through rationalization and other means. They are likely to feel a greater sense of control over their lives and have more confidence in achieving their goals (Ucar et al., 2019).

In addition, personal belief in a just world serves certain social functions. Individuals with a high personal belief in a just world often perceive themselves as being treated fairly and are motivated to take just actions. Their trust in justice fosters a sense of trust in their interpersonal relationships (Bègue, 2002). They are more likely to forgive rather than seek revenge; thus, individuals with a high personal belief in a just world are more inclined to engage in prosocial behavior and resist antisocial behavior (Bartholomaeus and Strelan, 2019).

The belief in a just world theory posits that individuals need to believe that the world they inhabit is just and orderly (Learner, 1980). This belief has a motivational function, prompting individuals to act justly in order to maintain the fairness of the world, actively adhere to social norms, and reduce deviant behavior. Additionally, the belief in a just world has a trust function, leading individuals to believe that others and they themselves have just destinies and that they can receive what they deserve, thereby reducing feelings of injustice and subsequently lowering the occurrence of problematic behaviors. According to resilience theory, if individuals are protected by positive factors, they can thrive even in the face of significant threats or severe adversity (Luthar et al., 2000). Internal cognition may play an important role in producing adaptive outcomes (Rutter, 2000). Lerner and Miller's (1978) just world theory posited that belief in a just world serves an adaptive function, and therefore it may act as a protective factor for those who have experienced adversity, preventing them from taking an antisocial path.

Some studies have indeed found a negative correlation between belief in a just world and antisocial behavior (Donat et al., 2012). Individuals with a strong belief in a just world tend to perceive events as reasonable, believing that good and evil will ultimately be rewarded. This belief has an adaptive function and can serve as an important psychological resource in people's daily lives (Lerner and Miller, 1978; Dalbert, 2001). Therefore, belief in a just world can serve as a negative predictive factor for internet addiction caused by depression and loneliness. If an individual believes that the world is just, this belief will motivate them to remain optimistic about the future and proactively adhere to just principles in pursuing their goals. Supported by this belief, individuals are more likely to invest in their long-term plans and firmly believe that their efforts will be rewarded. Conversely, when belief in a just world is threatened, individuals tend to opt for small immediate rewards. When the future is uncertain, people are more likely to prioritize immediate benefits (Zhou, 2013). Thus, internet addiction may also represent a relinquishment of expectations for the future in favor of pursuing immediate gratification. Based on this, we propose the hypothesis that belief in a just world has a negative predictive effect on internet addiction.

### 1.2 The moderating role of legal cognition

Simply examining the correlations between variables is insufficient; to better intervene in internet addiction, it is necessary to focus on the direct mechanisms of the influencing factors. While existing research has demonstrated the effectiveness of behavioral self-monitoring and related cognitive strategies in addressing internet addiction (Theopilus et al., 2024), the role of legal cognition in influencing internet addiction remains largely unexplored. Legal cognition is one of the key indicators in the process of individual legal socialization. Based on the characteristics of individual cognitive development and relevant theories of moral cognition development, legal cognition can be divided into abstract legal cognition and concrete legal cognition. Abstract legal cognition encompasses the understanding of the essence, values, and functions of law; that is, it addresses what law is, the utility of law for humanity, and how law meets human needs. Concrete legal cognition, on the other hand, refers to the awareness of current national laws, primarily involving the recognition of rights and obligations (Xu and Yan, 2022).

Could legal cognition play a moderating role in the relationship between belief in a just world and internet addiction? The cognitive-behavioral theory of internet addiction posits that it results from the interaction between proximal and distal factors. Distal factors include psychopathology and situational cues, while proximal factors refer to maladaptive cognition. Together, these factors constitute the sufficient conditions for the occurrence of internet addiction (Davis, 2001). The cognitive-behavioral model of pathological internet use indicates that cognitive characteristics and thought patterns among individual factors can influence internet addiction, with problematic cognition leading to persistent maladaptive responses. This theoretical model emphasizes that cognition or thinking is a primary cause of problematic behaviors, where maladaptive cognitions about oneself or the world are proximal factors that trigger and maintain internet addiction (King and Delfabbro, 2014). The belief in a just world theory asserts that individuals can gain a sense of control over their world only when they are willing to believe that the world is just, stable, and orderly, and that they will be treated fairly. This belief enables them to focus on long-term goals, invest resources into achieving these goals, and follow social norms to attain the outcomes they deserve (Hafer, 2000). Legal cognition pertains to the current judicial system of the state and its related activities, reflecting individuals' perceptions of the social control system. From this perspective, legal cognition can be considered a distal factor in internet addiction, representing individuals' awareness of the social context of their lives, while belief in a just world is considered a proximal cognitive factor. Based on this, we propose the hypothesis that legal cognition moderates the effect of belief in a just world on internet addiction.

## 2 Methods

### 2.1 Participants

The participants in this study were college students from universities in mainland China. A total of 532 questionnaires were distributed, and 409 valid responses were collected (76.88%). Among the respondents, there were 186 males and 223 females; 124 were freshmen, 55 sophomores, 125 juniors, and 105 seniors. This study employed a supervised computer-administered group testing protocol conducted in a controlled laboratory setting. Following informed consent procedures, participants accessed the study measures via a secure online platform distributed through institutional communication channels. The proctored testing session was completed within a standardized 15-min timeframe under direct researcher supervision, with robust safeguards ensuring data anonymity and confidentiality throughout all phases of data collection. The study received ethical approval from the Ethics Review Committee of Wenzhou University, ensuring that all procedures complied with ethical standards.

### 2.2 Measures

#### 2.2.1 Belief in a just world scale

The Chinese version of the Dalbert Belief in a Just World Scale, translated and revised by Chinese scholars, was utilized (Dalbert, 1999; Su et al., 2012). This scale consists of 13 items, divided into two subscales: the General Belief in Justice Scale includes 6 items, while the Personal Belief in a Just World Scale contains 7 items. A 6-point Likert scale was employed, ranging from “1 = strongly disagree” to “6 = strongly agree,” with all items scored positively. Higher scores indicate a stronger belief in a just world. In this study, the Cronbach's alpha coefficient was 0.93. The confirmatory factor analysis was conducted using maximum likelihood estimation. The results indicated that the model fit the data well, with the following fit indices: χ^2^/df = 2.73, NFI = 0.95, RFI = 0.93, IFI = 0.97, TLI = 0.96, CFI = 0.97, and RMSEA = 0.06.

#### 2.2.2 Internet addiction scale

The Internet Addiction Scale developed by Young (1998) was employed to measure the degree of individual internet addiction (Young, 2004). The scale encompasses six factors: salience, excessive use, neglect of work, anticipation, lack of control, and neglect of social life, comprising a total of 20 items. A 5-point Likert scale was utilized for scoring (1 = “strongly disagree,” 5 = “strongly agree”), with higher total scores indicating a greater tendency toward internet addiction. Specifically, scores ranging from 20 to 49 indicate normal internet usage, while scores of 50 or above signify internet addiction. In this study, the Cronbach's alpha coefficient was 0.94. Confirmatory factor analysis was conducted using maximum likelihood estimation. The results indicated that the model fit the data well, with the following fit indices: χ^2^/df = 3.57, NFI = 0.91, RFI = 0.89, IFI = 0.93, TLI = 0.92, CFI = 0.93, and RMSEA = 0.07.

#### 2.2.3 Legal cognition scale

This scale was adapted from the “Legal Cognition Scale” developed by Xu Shuhui and includes a total of 29 items across six dimensions: concrete legal cognition (constitutional cognition, rights cognition, obligations cognition) and abstract legal cognition (cognition of the nature of law, value cognition, cognition of the functions of law) (Xu, 2019). The scale employs a 5-point scoring system, where 1 indicates “strongly disagree” and 5 indicates “strongly agree,” with higher scores indicating a greater level of legal cognition. In this study, the Cronbach's alpha coefficient was 0.98. Confirmatory factor analysis was conducted using maximum likelihood estimation. The results indicated that the model fit the data well, with the following fit indices: χ^2^/df = 3.61, GFI = 0.94, IFI = 0.95, TLI = 0.93, CFI = 0.93, and RMSEA = 0.07.

### 2.3 Data analysis

SPSS 26.0 was used to conduct common method bias testing, descriptive statistics, and correlation analysis of the variables. Additionally, the PROCESS macro in SPSS was employed to test the moderating effect of legal cognition on the relationship between belief in a just world and internet addiction. Specifically, the significance of the moderating effect was first tested, followed by simple slope analysis to examine how different levels of legal cognition moderated the relationship between belief in a just world and internet addiction. Finally, the results of the moderating effect were presented visually through graphs.

## 3 Results

### 3.1 Common method bias

Common method bias was tested using Harman's single factor method. Factor analysis was conducted on all items of the collected data, revealing that there were 8 common factors with eigenvalues >1. The first factor accounted for 34.29% of the variance, which is below the 40% threshold. Therefore, the data in this study do not exhibit significant common method bias.

### 3.2 Descriptive statistics and correlation analysis

The results of the correlation analysis indicated that belief in a just world was significantly positively correlated with legal cognition, concrete legal cognition, and abstract legal cognition, while it was significantly negatively correlated with internet addiction. Internet addiction was not significantly correlated with legal cognition, concrete legal cognition, or abstract legal cognition. See Table 1 for details.

**Table 1:** Descriptive statistics and correlation analysis of variables.

**Variable**	**M ±SD**	**1**	**2**	**3**	**4**	**5**
1 BJW	3.45 ± 0.79	1				
2 LC	4.52 ± 0.62	0.30^**^	1			
3 CLC	58.90 ± 8.19	0.29^**^	0.99^**^	1		
4 ALC	72.12 ± 10.08	0.30^**^	0.99^**^	0.96^**^	1	
5 IA	2.15 ± 0.82	−0.17^**^	−0.34	−0.19	−0.45	1

^**^p < 0.01, BJW, belief in a just world; LC, legal cognition; CLC, concrete legal cognition; ALC, abstract legal cognition; IA, internet addiction.

### 3.3 Testing the moderating effect of legal cognition

The statistical analysis was conducted using standardized scores (*z*-scores) for all study variables, with subsequent examination performed through Model 1 of Hayes' PROCESS macro analysis. Belief in a just world was set as the independent variable, internet addiction as the dependent variable, and legal cognition as the moderating variable. Gender, age, parental education level, family income, and whether the participant was an only child were controlled. The results indicated that belief in a just world had a significant negative predictive effect on internet addiction, and the interaction term between belief in a just world and total legal cognition score significantly predicted internet addiction negatively. This suggests that total legal cognition served as a moderating factor between belief in a just world and internet addiction, as shown in Table 2.

**Table 2:** Moderating effect of legal cognition on the relationship between belief in a just world and internet addiction.

**Variable**	**Internet addiction**			
	β	**SE**	***t***	**95% CI**
Constant	0.45	0.21	2.14^*^	[0.04, 0.85]
BJW	−0.18	0.05	−3.56^***^	[−0.27, −0.08]
LC	−0.03	0.05	−0.56	[−0.14, 0.08]
BJW × LC	−0.08	0.04	−2.01^*^	[−0.16, 0.00]
Gender	−0.27	0.10	−2.86^**^	[−0.46, −0.09]
Age	0.07	0.04	1.88	[0.00, 0.15]
PEL	−0.05	0.03	−1.51	[−0.11, 0.01]
FI	−0.05	0.03	−1.72	[−0.11, 0.01]
WOC	−0.17	0.10	−1.65	[−0.36, 0.03]
*R^2^*	0.11			
*F*	5.87^***^			

^*^p < 0.05, ^**^p < 0.01, ^***^p < 0.001; BJW, Belief in a Just World ; LC, Legal Cognition; PEL, Parental education level; FI, Family income; WOC, Whether an only child.

**Figure 1:**
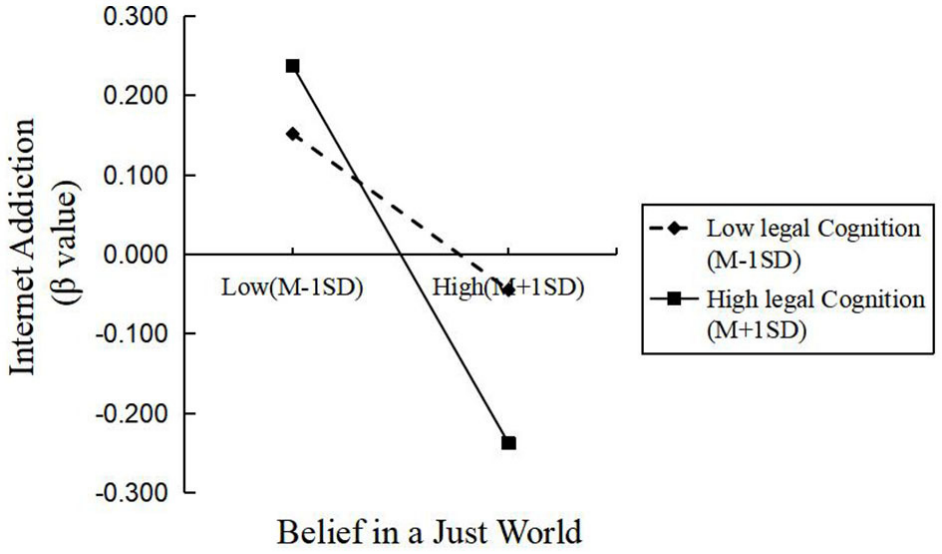
Moderating effect of legal cognition on the relationship between belief in a just world and internet addiction.

The standardized total legal cognition scores were divided into high and low groups based on one standard deviation from the mean, and simple slope analysis was conducted to explore the moderating effect, as shown in Figure 1. In the low group, the predictive effect of belief in a just world on internet addiction was not significant (β_simple = −0.09, *t* = −1.54, *p* > 0.05). In contrast, in the high group, belief in a just world had a significant negative predictive effect on internet addiction (β_simple = −0.23, *t* = −4.15, *p* < 0.001). The simple slope analysis showed that the relationship was not significant in the low-score group but became significant in the high-score group, indicating a relatively strong moderating effect.

Using the same method, the moderating effects of concrete legal cognition and abstract legal cognition were examined. The results indicated that belief in a just world had a significant negative predictive effect on internet addiction (β = −0.18, SE = 0.05, *t* = −3.59, *p* < 0.01). However, the interaction term between belief in a just world and concrete legal cognition was not significant in predicting internet addiction (β = −0.01, SE = 0.05, *t* = −1.80, *p* = 0.07). This suggests that concrete legal cognition does not moderate the relationship between belief in a just world and internet addiction.

Belief in a just world had a significant negative predictive effect on internet addiction, and the interaction term between belief in a just world and abstract legal cognition also significantly predicted internet addiction negatively. This indicated that abstract legal cognition moderated the relationship between belief in a just world and internet addiction. The results were presented in Table 3.

**Table 3:** Moderating effect of abstract legal cognition on the relationship between belief in a just world and internet addiction.

**Variable**	**Internet addiction**			
	β	**SE**	***t***	**95% CI**
Constant	0.45	0.21	2.16^*^	[0.04, 0.86]
BJW	−0.17	0.05	−3.51^***^	[−0.27, −0.08]
ALC	−0.04	0.05	−0.82	[-0.15, 0.06]
BJW × ALC	−0.09	0.04	−2.25^**^	[−0.17, −0.01]
Gender	−0.27	0.10	−2.86^**^	[−0.46, −0.09]
Age	0.07	0.04	1.86	[0.00, 0.15]
PEL	−0.05	0.03	−1.53	[−0.11, 0.01]
FI	−0.05	0.03	−1.70	[−0.11, 0.01]
WOC	−0.17	0.10	−1.64	[−0.36, 0.03]
*R^2^*	0.11			
*F*	6.01^***^			

^*^p < 0.05, ^**^p < 0.01, ^***^p < 0.001; BJW, Belief in a Just World ; ALC, Abstract legal Cognition; PEL, Parental education level; FI, family income;WOC, Whether an only child.

**Figure 2:**
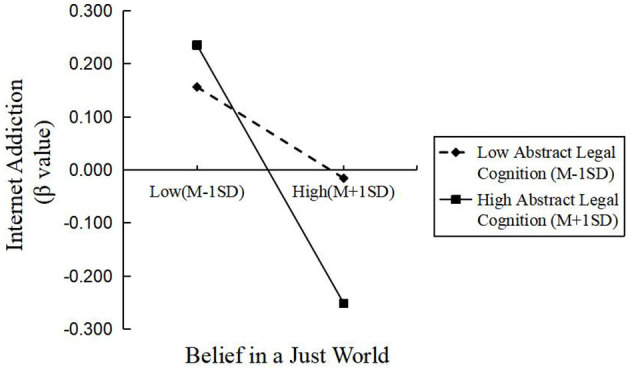
Moderating effect of abstract legal cognition on the relationship between belief in a just world and internet addiction.

The standardized scores of abstract legal cognition were divided into high and low groups based on one standard deviation from the mean, and simple slope analysis was conducted to explore the moderating effect, as shown in Figure 2. In the low group, the predictive effect of belief in a just world on internet addiction was not significant (β_simple = −0.08, *t* = −1.34, *p* > 0.05). In contrast, in the high group, belief in a just world had a significant negative predictive effect on internet addiction (β_simple = −0.24, *t* = −4.23, *p* < 0.001). The simple slope analysis showed that the relationship was not significant in the low-score group but became significant in the high-score group, indicating a relatively strong moderating effect. This indicated that as the level of abstract legal cognition increased, the negative predictive effect of belief in a just world on internet addiction became significant.

## 4 Discussion

This study found that belief in a just world significantly negatively predicted internet addiction among adolescents, indicating that individuals' belief in a just world serves as an important protective factor against youth internet addiction. This finding is consistent with previous research, which identified a negative predictive effect of belief in a just world on externalizing problems such as criminal behavior and aggression (Kong et al., 2021). Belief in a just world was negatively correlated with antisocial behavior (Kong et al., 2021). This suggests that internet addiction shares similar underlying mechanisms with these problematic behaviors. The integration model of personality-emotion-cognition-execution posits that in the early stages, individual core characteristics serve as susceptibility factors for addiction, such as genetics, temperament, coping styles, motivations, and values. These traits drive individuals to satisfy their needs through gaming. As individuals repeatedly fulfill their needs through this behavioral pattern, it can lead to dependency on the internet, such as frequently using games to alleviate negative emotions. In this process, their control systems are compromised, leading to impulsive behaviors based on immediate gratification (Brand et al., 2016, 2019). When the impulsive system dominates, individuals enter the late stages of internet addiction, where the addictive behavior becomes automated (Nie, 2022). Group counseling using cognitive behavioral therapy strategies has found that students can change their internet usage patterns and engage in healthier online behaviors after treatment (Saroinsong, 2019). Previous research indeed showed that individual characteristics (such as personality, cognition, and emotion) were influencing factors in internet addiction (Kökönyei et al., 2019). As an individual cognitive characteristic, belief in a just world predicted internet addiction. Additionally, the mechanisms by which belief in a just world promotes mental health can be explained through two hypotheses: one as a psychological resource and the other as a psychological buffer. The former suggests that activating belief in a just world leads to increased life satisfaction, which in turn enhances belief in a just world (Correia et al., 2009). The latter indicates that individuals with higher belief in a just world exhibit lower levels of anger in provoking situations, without a corresponding decrease in self-esteem. Conversely, those with relatively low belief in a just world display greater anger and experience a decline in self-esteem (Dalbert, 2002). Consequently, belief in a just world not only exerts an influence on internet addiction but also mitigates the propensity for such addiction by offering positive psychological resources and promoting adaptive cognitive frameworks.

The negative correlation between internet addiction and belief in a just world suggests that individuals with a stronger belief in a just and orderly world are more likely to maintain rational behavior, adhere to social norms, and consequently reduce excessive dependence on the virtual world. The practical significance of this finding lies in its potential application as an effective strategy for preventing and intervening in internet addiction by enhancing individuals' belief in a just world, thereby increasing psychological satisfaction in real life and reducing the tendency to escape into the virtual world. In the context of adolescent internet behavior education, fostering a belief in a just world can contribute to the development of positive social cognition, improve self-regulation, and mitigate the risk of internet addiction. Moreover, promoting core values such as fairness and justice can strengthen individuals' trust in social order, decreasing the inclination to seek solace in the virtual world due to dissatisfaction with reality. These findings provide a theoretical foundation for internet governance and policy-making, further contributing to social harmony and the healthy development of cyberspace.

This study further examined the mechanisms through which belief in a just world influences internet addiction. The findings revealed that the overall level of legal cognition and abstract legal cognition significantly moderated the effect of belief in a just world on internet addiction, whereas concrete legal cognition did not have a moderating effect on this relationship. Specifically, compared to individuals with low legal cognition (abstract legal cognition), the negative predictive effect of belief in a just world on internet addiction was significant for individuals with high legal cognition (abstract legal cognition). In other words, an increase in legal cognition (abstract legal cognition) strengthened the negative predictive effect of belief in a just world on internet addiction.

The theory of justice motivation posits that individuals with a strong belief in a just world, upon feeling fairly treated, are more likely to attribute it to the goodness of others and believe they should reciprocate by helping others (Jiang et al., 2016; Schindler et al., 2019). Individuals who hold a belief in a just world perceive the world as orderly and fair, believing that people will receive their due rewards or punishments (Lerner and Miller, 1978). This belief provides individuals with a sense of security and control, motivating them to pursue long-term goals and adhere to social norms. Legal cognition encompasses an understanding of the nature, values, and functions of law, as well as rights and obligations. Individuals with high levels of legal cognition, particularly those with strong abstract legal reasoning, recognize that law serves to maintain social order and promotes the welfare of all humanity. According to Media-System Dependency theory, the greater an individual's reliance on a particular medium, the greater its influence on that individual. When individuals feel threatened in their real-life environment, they may develop a need to escape from reality, leading them to rely on the internet (Huber et al., 2022). However, individuals with a high level of legal cognition can rely on the national judicial system to cope with real-life difficulties, rather than turning to the online world. Therefore, individuals with high legal cognition (particularly abstract legal cognition) are less likely to resort to negative coping mechanisms, such as becoming addicted to the internet to alleviate stress, even when faced with challenges, as they maintain a belief in a just world. Related research found that there is a relationship between personal belief in a just world and rule-breaking behavior (Correia and Dalbert, 2008). For example, researchers found in a sample of young offenders that adolescents with a stronger personal belief in a just world exhibited fewer disciplinary issues compared to those with a weaker belief. In other words, young individuals with a high belief in a just world are more likely to perceive legal procedures as fair, leading them to trust in the fairness of the state and, consequently, motivating them to comply with the law (Otto and Dalbert, 2005). Simultaneously, dual-process theory is often utilized to explain the underlying mechanisms of addiction. According to this framework, an individual's cognitive processes and decision-making are facilitated by the interplay between automatic processing systems and controlled processing systems (De Neys and Glumicic, 2008). Research has found that the development of addictive behaviors is associated with an enhancement of the automatic processing system and a weakening of the controlled processing system (Lindgren et al., 2019). The philosophical foundation of the law's controlling function in society is based on the view of individuals as reasonable persons. The characteristic of a reasonable person is the ability to take responsibility for their actions and make rational decisions. Therefore, individuals with a high level of legal cognition have their behavior and decision-making predominantly guided by the controlled processing system. As a result, this can inhibit addictive behaviors related to the internet. Additionally, according to the reciprocal enhancement model of the “protective factor-protective factor model,” one protective factor can amplify or enhance the facilitating effect of another protective factor on individual development (Fergus and Zimmerman, 2005). Both belief in a just world and legal cognition serve as protective factors against internet addiction, with one factor potentially enhancing the inhibitory effect of the other on the development of internet addiction.

However, this study found that concrete legal cognition did not moderate the relationship between belief in a just world and internet addiction. The reasons for this may be attributed to several factors. On one hand, concrete legal cognition pertains to an individual's understanding of the current laws and related systems of the state, primarily focusing on rights and obligations. In China, the traditional model of social governance is characterized by a stability-control approach that suppresses expressions of public interest. In this context, power often exceeds its boundaries, encroaching upon the private sphere and constraining individual rights (Pan and Cai, 2013). This may lead to a lack of proactive understanding of rights and obligations among adolescents, thereby hindering the strengthening effect of just world beliefs on the impact of internet addiction. On the other hand, the understanding of rights and obligations among Chinese adolescents is primarily promoted through legal education in schools. However, there are several practical challenges to the high-quality development of legal education for adolescents in China, such as a lack of qualified teachers, insufficient development of legal education resources, and an inadequate evaluation system for legal education (Li and Zheng, 2024). This, to some extent, affects the development of adolescents' concrete legal cognition, thereby failing to enhance the inhibitory effect of belief in a just world on internet addiction.

Based on the moderating role of legal cognition in internet addiction, this paper proposes the following practical applications and intervention strategies: First, by enhancing adolescents' level of legal cognition, their self-restraint can be strengthened, reducing the likelihood of internet addiction. Specifically, schools can integrate legal cognition education into their curricula, employing interactive teaching methods, case analyses, and mock court sessions to help adolescents understand the value of law in real-world contexts, thereby improving their legal cognition. Secondly, by combining psychological interventions with the enhancement of legal cognition, cognitive-behavioral interventions (CBI) can be designed to assist adolescents in making rational decisions when facing online temptations, thereby enhancing their self-regulation in the digital environment. Furthermore, at the family level, improving parents' legal cognition can further strengthen the supervision and guidance of adolescents' internet behaviors, forming a collaborative intervention mechanism between the family and the school. Finally, at the societal level, cooperation among the government, communities, and schools should be encouraged to promote the widespread adoption of legal cognition education, alongside the development of internet behavior regulations, to provide comprehensive legal protection and support for adolescents. These intervention measures not only help prevent internet addiction but also assist adolescents in cultivating healthy internet usage habits, promoting their overall physical and mental wellbeing.

Existing research indicates that early explorations of legal socialization were primarily based on cognitive development and rational models to explain individuals' acceptance or violation of rules and their relationship with criminal or recidivist behavior (Penner et al., 2014; Trinkner and Cohn, 2014). These studies emphasize that cognitive factors in the legal socialization process play a critical role in shaping individuals' compliance with or deviation from legal and social norms. However, although problematic internet use is closely associated with bullying and criminal behavior among children and adolescents—and excessive internet use not only affects mental health but also increases the likelihood of engaging in high-risk digital behaviors—there has been limited attention to the role of legal socialization factors in this context. This study addresses this gap by introducing legal cognition, a key indicator of legal socialization, to examine its impact on problematic internet use. By incorporating legal cognition as a novel variable, this study extends the application of legal socialization theory to the digital age and provides new theoretical insights and empirical evidence for understanding and addressing internet addiction.

## 5 Limitations and suggestions for future research

First, this study employed a cross-sectional design using a questionnaire method, which is insufficient for fully capturing the long-term dynamic relationships between variables. Therefore, future research could adopt a longitudinal design to explore the long-term impact mechanisms of belief in a just world on internet addiction, thereby providing more accurate validation of causal relationships between variables. Additionally, to avoid relying solely on quantitative methods, future research could integrate qualitative approaches (such as interviews or focus groups) to gain deeper insights into the psychological mechanisms and social influences underlying internet addiction among college students.

Second, the data in this study were obtained through self-reporting, which may be subject to social desirability effects and subjective cognitive biases. Future research could utilize multi-source data collection (e.g., peer assessments, teacher evaluations, or behavioral data) to mitigate biases associated with self-reporting, thereby enhancing the reliability of the data and the external validity of the findings.

Third, this study examined legal cognition as a moderating factor, considering it a situational factor. However, this measurement approach may not fully capture individuals' genuine responses in specific legal contexts. Therefore, future research could create more realistic legal scenarios (such as virtual simulations or situational experiments) to dynamically assess legal cognition in various legal environments, providing a more comprehensive understanding of its moderating effects on individual behavior.

Future research suggestions are as follows:

First, adopt a longitudinal research design. Future studies could collect data at multiple time points to track the long-term developmental trajectory of belief in a just world, legal cognition, and internet addiction, and examine the causal relationships between these variables.

Second, utilize multi-source data collection. Future research is encouraged to incorporate behavioral data, peer evaluations, and experimental data to comprehensively examine the relationships between variables from a multidimensional perspective, thereby enhancing the robustness of the findings.

Third, contextualize the measurement of legal cognition. Future studies could employ experimental paradigms or virtual reality technology to create various legal scenarios and dynamically assess individuals' legal cognition, thereby further validating its moderating effect on internet addiction.

Fourth, conduct cross-cultural comparative research. Existing studies suggest that internet addiction is closely related to mental health, particularly in terms of anxiety, depression, alexithymia, and emotional dysregulation, which may be more pronounced in certain vulnerable populations (Liu W. et al., 2025; Yi et al., in press; Liu X. et al., 2025). Although this study focused on college students, the impact of internet addiction on the mental health of different populations is also of great significance and warrants further exploration. Therefore, future research could conduct cross-cultural comparisons in different cultural contexts to examine the universality and variability of legal cognition across cultural groups, thereby enhancing the generalizability of the findings.

## 6 Conclusions

This study introduces legal cognition as a novel variable in understanding the mechanisms of internet addiction, thereby enriching the theoretical research surrounding this topic. The findings indicate that belief in a just world negatively predicts internet addiction, suggesting that cognitive therapies targeting individual beliefs could effectively modify addictive behaviors. Furthermore, this research reveals that legal cognition, particularly abstract legal cognition, plays a reinforcing role in the relationship between belief in a just world and internet addiction. Therefore, prevention and intervention strategies for internet addiction could focus on enhancing legal education in schools, emphasizing the understanding of the value and function of law and its systems among college students.

## Data Availability

The raw data supporting the conclusions of this article will be made available by the authors, without undue reservation.
